# When grooming stalls: Assessing communicative repair, negotiation, and progressivity in chimpanzees

**DOI:** 10.1016/j.isci.2026.117495

**Published:** 2026-09-23

**Authors:** Kayla Kolff, Simone Pika

**Affiliations:** 1 Comparative BioCognition, Institute of Cognitive Science, Osnabrück University, Osnabrück, Lower Saxony, Germany; 2 Ozouga Chimpanzee Project, Loango National Park, Iguéla, Gabon

**Keywords:** *Pan troglodytes*, communication, gestures, social interactions, turn-taking, cooperation

## Abstract

Human social interaction is built on progressivity (the forward flow of interaction), which communicative breakdowns may disrupt. Communicative repair (resolving communicative troubles) and negotiation (a collaborative sequence to resolve communicative breakdowns) may both serve as processes to restore progressivity following breakdowns. Here, we investigated gestural redoings(reproduction of a previous signal in any form), negotiation, and progressivity in grooming interactions (*N* = 311) among male East African chimpanzees (*Pan troglodytes schweinfurthii*) in the wild. These chimpanzees produced gestural redoings as repetitions, substitutions, and modifications (*N* = 158) occurring at delayed temporal gaps relative to grooming turn-taking latencies. Both substitutions and negotiations (*N* = 219) were associated with restored progressivity. Strongly bonded pairs showed a higher likelihood of compromise outcomes during negotiation. These findings indicate that these chimpanzees employed repair-like processes associated with restored progressivity, bearing certain resemblances to those observed in human conversation—raising the possibility that such capacities may have evolutionary roots predating the emergence of language.

## Highlights

• Chimpanzees' gestural redoings were delayed relative to reported turn-taking latencies
• redoings and negotiations were generally associated with restored progressivity
• Social bonds influenced negotiation outcomes
• redoings and negotiations seemingly share features with human communicative repair

## Graphical abstract

**Figure:**
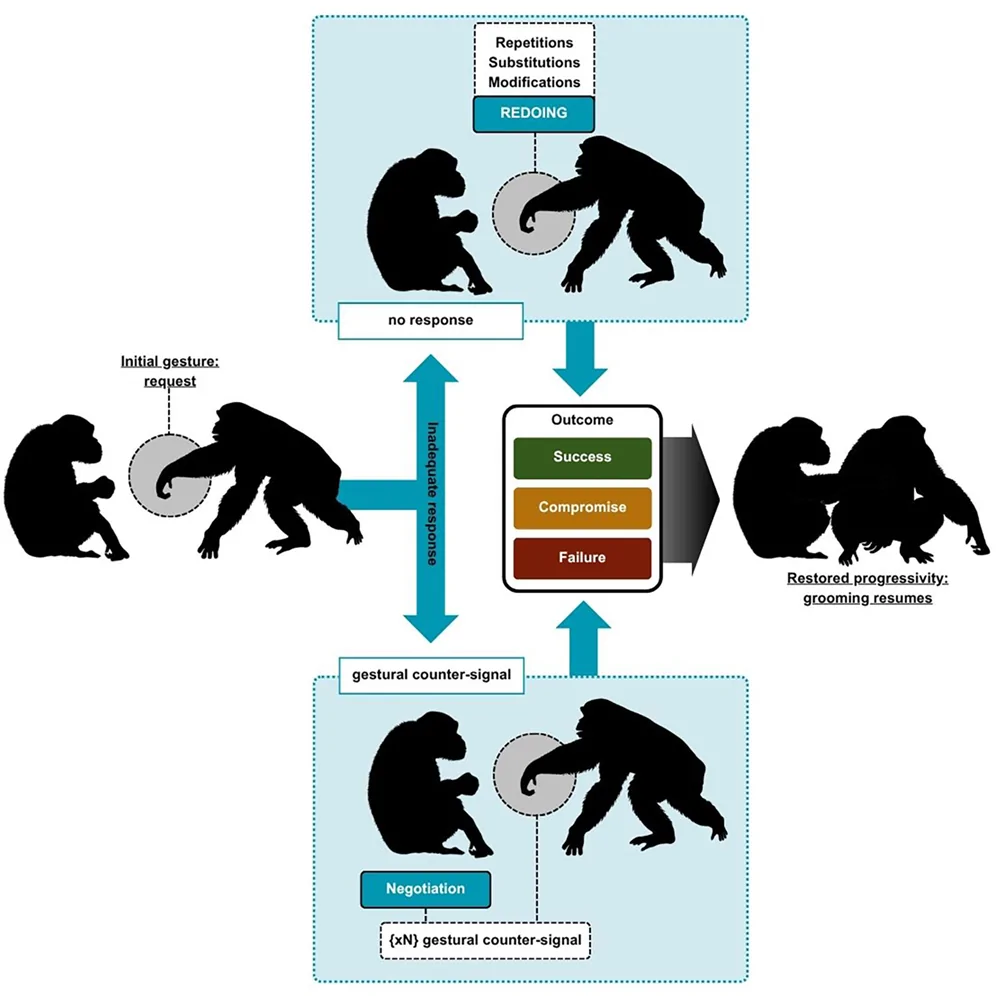

## Teaser

Communication; Animal behavior; Zoology

## Introduction

Human social action is inherently cooperative,^1^ characterised by joint efforts to sustain interactional progression facilitated through communication. Progressivity, the forward movement of an interaction toward the completion of an activity,^2^ can be disrupted by communicative breakdowns, for example, miscommunication. Interactants may detect and resolve such breakdowns through a process known as communicative repair,^3^ first defined within conversation analysis,^3^^,^^4^ which remains a prominent approach to studying human social interaction.^5^ Communicative repair can occur on the speaker’s own initiative (i.e., self-initiated repair; e.g., “She left on Thursday – err, Friday”)^3^ or in response to a recipient’s prompt or explicit signalling of misunderstanding (e.g., a listener asking “Thursday or Friday?”). The latter constitutes other-initiated repair,^6^ also termed interactive repair.^7^ A related but distinct process for restoring progressivity is negotiation of meaning (hereafter negotiation), a sequence in which interactants collaboratively work to resolve an actual or potential breakdown, during which the ongoing interaction is put on hold.^8^ For instance, this occurs in situations where individuals share a common goal but have their own ideas about how it should be pursued, as seen in peer play, where children constantly negotiate details until agreement is reached and play can continue.^9^ Both communicative repair and negotiation thus serve as processes to establish progressivity when breakdown arises,^10^^,^^11^ with other-initiated repair^12^ and negotiation in particular depending on the collaborative contributions of both interactants.

Communicative repair occurs across languages, with both self-initiated and other-initiated forms well documented.^3^^,^^13^ It can take several forms, including repeating a troubled source^14^^,^^15^^,^^16^^,^^17^ (i.e., *repetitions*), substituting the troubled source with a different communicative unit^18^^,^^19^ (i.e., *substitutions*), or modifying the troubled source by adding or deleting units^17^^,^^20^^,^^21^ (i.e., *modifications*). Beyond its forms, in spoken English, one study found other-initiated repair involving a delayed temporal gap of 700 ms,^22^ considerably longer than the approximately 100–300 ms typical of conversational turn-taking (the rapid and flexible exchange of information between interactants^4^).^22^^,^^23^ However, whether this delay is universal remains an open question.

Communicative repair has further been documented early in development,^24^ including self-initiated repair prompted by others at approximately two years of age.^25^ Similarly, negotiation has been observed from early infancy, as seen in pre-verbal infants (aged 11–18 months) already participating in back-and-forth exchanges following communicative failure, where mothers help infants clarify their communicative intentions.^19^ The early emergence of both communicative repair and negotiation suggests that these may represent fundamental interactional capacities that predate linguistic communication. This view is consistent with the “Interaction Engine” hypothesis, positing language as an adaptation built upon a set of interaction principles (i.e., turn-taking, sequence organisation, and communicative repair) and underlying cognitive capacities, such as intentionality and the ability to recognise and respond to others’ intentions.^26^^,^^27^ Negotiation, drawing on communicative repair and sequence organisation, may thus represent an equally fundamental feature of human interaction. If negotiation, like communicative repair, is among the building blocks of the Interaction Engine, we might expect to find precursors of both in nonhuman species, particularly our closest living relatives.

Evidence for self-initiated repair has been captured as signal redoings, defined as instances where a previous signal is produced again in any form^12^ (Table 1). redoings have been documented in nonhuman species, primarily in nonhuman primates, ranging from large-bodied apes^31^^,^^32^^,^^33^ to small-bodied apes^34^ and African monkeys.^35^^,^^36^ Negotiation (back-and-forth signalling, Table 1) has similarly been observed in nonhuman primates, but it has predominantly been examined in the context of gestural acquisition^37^^,^^38^ and resource conflict.^39^ Other-initiated repair, in which a recipient actively prompts the signaller to clarify a previous utterance, however, has been argued to be uniquely human, requiring the unique reflexivity of language.^7^ Existing methodological limitations, such as the reliance on experimental paradigms, often with human interactants, and a tendency to focus narrowly on the signaller while overlooking recipient responses,^12^^,^^40^^,^^41^ limit the extent to which this claim can be assessed. This limitation is compounded by terminological inconsistencies across studies: elaboration encompasses substitution, modification, or the use of multiple signals, while persistence variably covers repetitions or both repetitions and elaboration.^42^ There is therefore a lack of detailed and naturalistic analyses of communicative breakdown and repair that consider both interactants in nonhuman interactions and the recipient’s role in particular. Consequently, the role of negotiation in joint action and progressivity, and its relation to communicative repair in nonhuman primates, remain largely unaddressed.

**Table 1:** Operationalizations of terms used in this study.

Term	Operationalization
Communicative repair	Fixing of a communicative breakdown or trouble by a signaller (self-initiated) or recipient (other-initiated or interactive repair), which may lead to the restoration of progressivity
Gestures	Movements of the extremities, head, or body postures that could be directed toward a social partner^28^; they are mechanically ineffective, meaning they are produced to request or reach a goal, require active participation from the interactant, and potentially elicit a response from the recipient.^28^
Communicative breakdowns	Situations within any point of a grooming interaction where a gestural request by either interacting individual did not receive an apparently satisfactory outcome^12^ within a 30-s interval (time point categorising the end of a grooming interaction)^29^^,^^30^
redoings	Any instance in which a communicative signal is repeated, substituted, or modified following the absence of a desired response or a signal of trouble (i.e., communicative breakdown).
Repetition	Signaller reproduces the prior gesture or gestural sequence.
Substitution	Replacing a prior gesture or gestural sequence with a new one.
Modification	Combining another gesture with the prior gesture or reproducing a prior gestural sequence and excluding certain gestures.
Negotiation	Recipient producing a gesture in response to the signaller’s gestural request, which may lead to further negotiation (signaller counter-signals, and the recipient may counter-signal again), leading to more than one counter-signal; these can involve redoings (signaller produces aredoing, recipient counter-signals, or recipient counter-signals and redoes their gesture, producing a redoing).
Progressivity	Forward movement of an interaction toward the completion of an activity.^2^
Outcome	Categorised as success, compromise, failure, and unclear based on whether the presumed was met on the basis of the gestural meaning; success entailed when the recipient’s response met the presumed goal; compromise referred to cases where the response did not meet the presumed goal but the grooming interaction nevertheless continued; failure included when the recipient responded but the response did not meet the presumed goal and the interaction ceased; unclear was coded when the presumed goal could not be determined within the grooming context.
Restoration of progressivity	A success or compromise outcome of a redoing and negotiation.

Negotiation, while not constituting communicative repair itself, may fit within this communicative context. Unlike redoings, which only involve the signaller, it involves bilateral signalling emerging from communicative breakdown in which a counter-signal constitutes an inadequate response. It has the potential to restore progressivity, akin to other-initiated repair,^43^^,^^44^ and may draw on the interactional capacities underpinning it (e.g., self-and-other monitoring, interactional contingency, and flexibility in signalling^12^). In particular, cases involving the signaller producing a counter-signal in response to the recipient’s counter-signal (i.e., two counter-signals) may show parallels to third-turn repair. This is a form of self-initiated repair in which a speaker returns in the third turn to repair a troubled source from their original utterance, following a response from a recipient^45^^,^^46^ (e.g., speaker: “She left on Thursday”; listener: “Okay great”; speaker: “Or I mean Friday”). The gestures employed in these cases are the same as, or share a similar goal to, the signaller’s initial gestural request. As such, negotiation may offer a window into the evolutionary origins of other-initiated repair, potentially rooted in earlier interactional exchanges, shifting toward more collaborative forms with the development of language.

The present study aimed to increase the current understanding of the evolutionary trajectory of communicative repair and restoration of progressivity following communicative failure. To do so, we examined gestural redoings, negotiation, and their outcomes (Table 1) within the context of grooming—defined as manual parting and cleaning of another’s fur or skin to remove dirt and parasites,^47^ and essential in the establishment and maintenance of social bonds.^48^ The study was conducted on males of two East African chimpanzee (*Pan troglodytes schweinfurthii*) communities from the Kibale National Park, Uganda. We focused on chimpanzees because, alongside bonobos (*Pan paniscus*), they are our closest living relatives and provide an important model for reconstructing the socio-cognitive abilities of the last common ancestor.^49^ We selected grooming interactions because they share several features with human conversations^4^^,^^23^: they are frequent,^48^ requires moment-to-moment coordination over extended periods,^50^^,^^51^ involves reciprocity,^29^^,^^52^ and is supported by extensive communication.^53^^,^^54^ Grooming can occur over prolonged durations and involve one or multiple role reversals, requiring coordination between the groomer and groomee.^50^^,^^55^ This coordination is facilitated through gestures, which function as imperative requests^53^^,^^56^ and are considered the main channel of communication in such close-range interactions.^57^ Gestures therefore served as our primary focus, as communicative breakdowns may naturally arise when requests do not receive a satisfactory response (Table 1) and may be resolved through redoings and negotiation, making grooming an ideal setting to study these processes and their outcomes.

We focused on communicative breakdowns identified as gestural requests lacking the desired outcome,^12^ since this bottom-up approach does not require assessing misunderstandings or mind-reading. Doing so would require assessing perspective-taking (the ability to know what others know^58^), a capacity that is challenging to study in species living in the wild, often indirectly inferred,^59^^,^^60^ and whose presence in chimpanzees remains a topic of ongoing discussion.^58^^,^^61^^,^^62^ Therefore, we first identified communicative breakdowns during grooming interactions to establish cases where progressivity was disrupted. We subsequently assessed the presence of redoings (repetitions, substitutions, and modifications) and negotiation (back-and-forth gestural production between individuals), and examined the extent to which these were associated with the subsequent restoration of progressivity (Table 1; see Figure S1 for illustration), analogous to their role in human interaction.^11^^,^^45^ Additionally, we investigated the temporal dynamics of redoings relative to latencies established for turn-taking in chimpanzees^63^^,^^64^ to explore whether delayed timings, as observed in a study on human other-initiated repair,^6^ also characterise chimpanzee repair processes. Finally, given the well-documented role of social bonds in shaping grooming interactions,^65^ particularly among male chimpanzees, we considered their potential influence on the use of redoings, negotiation, and the resulting outcomes, as social dynamics may shape the presence and function of these interactional processes.^31^

We hypothesised that (1) redoings and negotiation in chimpanzees represent structural processes that share features with communicative repair (H1), (2) redoings occur with delayed timings that do not differ across repetitions, substitutions, and modifications (H2), (3) redoings and negotiation restore progressivity (H3), and (4) the production of redoings, negotiation, and their outcomes is influenced by the strength of the social bond between the interactants (H4).

## Results

We analysed video footage of 311 grooming interactions from 157 dyads across 42 adult and subadult males to investigate the presence of redoings and negotiations during breakdowns (see STAR Methods for coding details). From these interactions, 144 included 158 redoings (1.88 ± 1.45 per interaction; 2.43 ± 2.15 per dyad) and 219 negotiations (1.89 ± 1.65 per interaction; 2.84 ± 3.38 per dyad) which also involved 15 redoings.

### Repetition and substitution characterised redoings, while negotiations primarily involved one counter-signal

To test the first hypothesis (H1), whether redoings and negotiation represent structural processes that share features with human communicative repair, we examined the processes underlying each type (Table 2). For this, we looked into communicative breakdowns and the processes that followed. We found that repetitions and substitutions accounted for the majority of redoings, while negotiations were predominantly one-counter-signal exchanges (Table 2). Of the negotiations involving more than one counter-signal, 23 included an interactant substituting their previous gesture (18 [78%] same presumed goal and 5 [22%] different goal), and 11 repeating it in the next turn. The results demonstrated the presence of redoings and cases of negotiation with two or three counter-signals involving the repetition of gestures or substitution of gestures for the same goal. These processes are seemingly similar to those described in human communicative repair, particularly self-initiated repair, consistent with the hypothesis that comparable processes in chimpanzees share structural features.

**Table 2:** Summary of redoings and negotiations

	Frequency (*N*°)		Timing (s)		Number of outcomes			
	Total	redoings	Median	Mean	Success	Compromise	Failure	Unclear
redoings	158	–	0.38	1.31 ± 2.57	32	64	60	2
Repetitions	77	–	0.43	1.68 ± 3.14	13	26	37	1
Substitution	74	–	0.23	0.96 ± 1.87	18	37	18	1
Modification	7	–	0.35	0.85 ± 0.84	1	1	5	–
Negotiation	219	15	–	–	103	98	13	5
One counter-signal	188	12	–	–	96	77	10	5
Two counter-signals	28	3	–	–	4	21	3	–
Three counter-signals	3	0	–	–	3	0	0	–

### redoing latencies did not vary by type but were delayed relative to gesture-response pairs

To investigatethe second hypothesis (H2), that redoings occur with delayed timings that do not differ across types, we examined the temporal gap latencies. We measured the timing from the offset of the prior gestural signal to the onset of the following gestural signal produced by the same signaller, log-transformed the resulting values and ran a Bayesian generalised mixed model (see STAR Methods for full description of the model). Temporal analyses showed that there was no evidence of latency differences between the redoings (all 89% credible intervals included zero, see Figure 1 and Tables 2 and S3). This result supported the hypothesis that redoing latencies were not credibly different across repetitions, substitutions, and modifications, but occur with delayed timings relative to gesture-response turn-taking (median: -0.06 s^63^; Figure 1).

**Figure 1:**
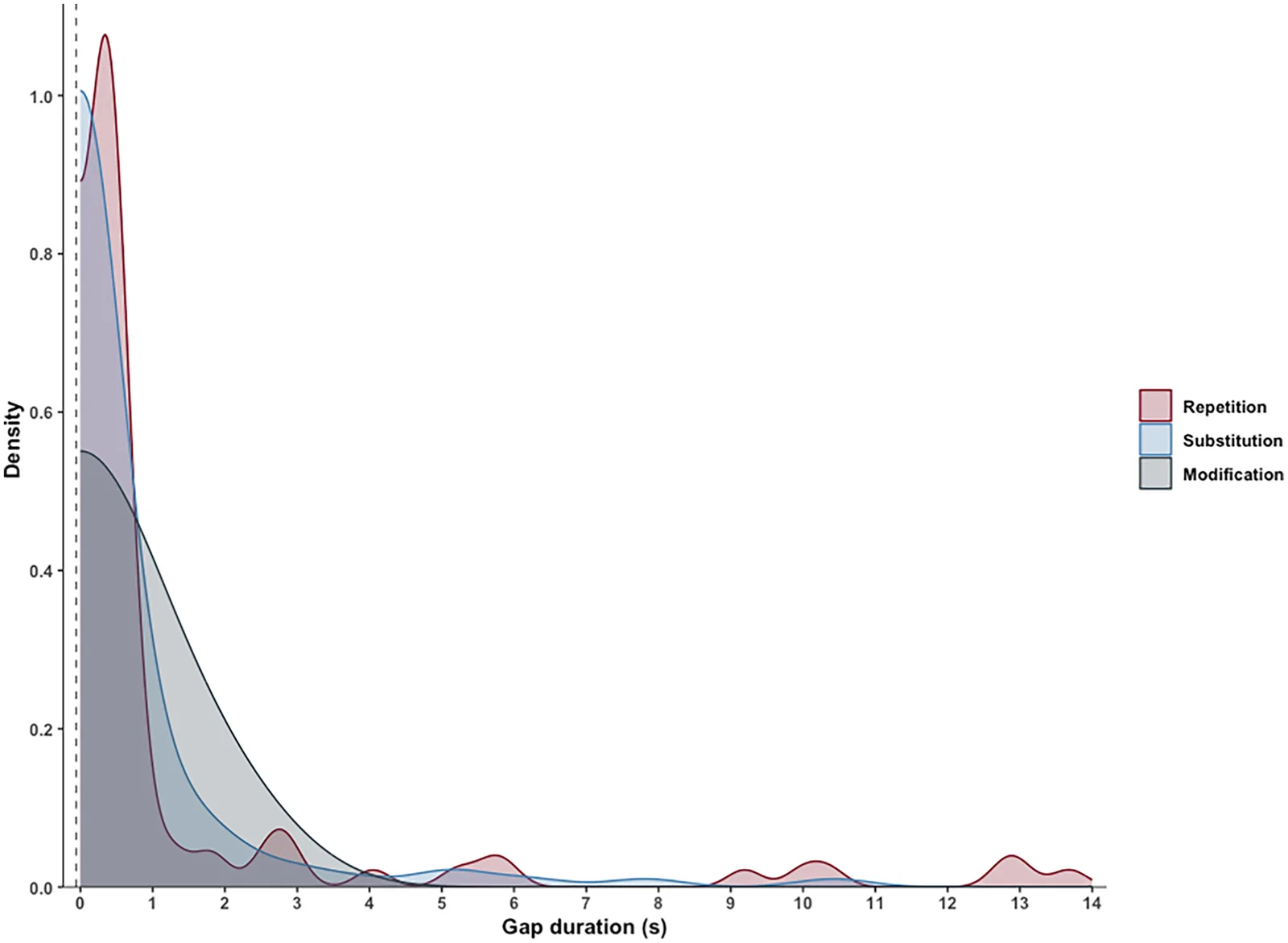
Distribution of temporal gap latencies (s) of each redoing Density curves are shown per redoing. The gray vertical dashed line indicates the latency found for gesture-response turn-taking in grooming^63^ (i.e., request-grant pairs^66^).

### redoings and negotiations predominantly led to restored progressivity

To test the third hypothesis (H3), we examined the outcomes of redoings and negotiation to assess progressivity restoration. To assess progressivity, we investigated whether the last gestural signal in each redoing or negotiation sequence resulted in the presumed goal being met (i.e., an apparent satisfactory outcome, see Table 3). Outcomes were categorised as success (goal met), compromise (goal not met, but grooming continued), failure (goal not met and grooming did not continue), or unclear (goal unknown within the grooming context). Success and compromise outcomes were classified as restored progressivity. Concerning redoings, compromise was the most common outcome, followed by failure and success (Table 2). Failure was the most common outcome for modifications and repetitions, while compromise was the predominant outcome for substitutions (Table 2). For negotiations, outcomes were most frequently success or compromise and varied by the number of counter-signals: one-counter-signal exchanges were most often successful, two-counter-signal exchanges predominantly resulted in compromise, and the rare three-counter-signal exchanges all resulted in success (Table 2). The results support the hypothesis that redoings and negotiations restore progressivity, as overall redoings (61%) and negotiations (92%) predominantly led to restored progressivity from the number of success and compromise outcomes. However, this outcome varied by process—failure was more common for modifications and repetitions, compromise predominated for substitutions, and success was more likely with fewer counter-signals in negotiations.

**Table 3:** Gestures and their presumed goals in the grooming context based on previous studies

Gesture	Presumed goal
bitekiss	Grooming solicitation (request to be groomed).^53^
exaggerated loud scratch	Grooming solicitation (request to be groomed).^53^^,^^54^^,^^56^
extend limb	Grooming solicitation (request to be groomed).^53^
grab-pull	Reposition body (move body into indicated position).^53^
grab hold	Grooming solicitation (request to be groomed), reposition body (move body into indicated position).^92^
leaf groom	Unclear.
peer	Unclear.
present	Grooming solicitation (request to be groomed).^53^^,^^54^
push	Reposition body (move body into indicated position).^53^^,^^54^
raise limb	Grooming solicitation (request to be groomed).^53^
touch	Reposition body (move body into indicated position).^53^
touch hold	Reposition body (move body into indicated position).^53^

### Stronger social bonds decreased negotiation success relative to compromise outcomes, but showed no effect on redoings

To investigate the fourth hypothesis (H4), we examined whether redoings, negotiation, and their outcomes were influenced by the social bond strength between the interacting individuals. We calculated the social bond strength using a Dyadic Sociality Index,^67^ where dyads with higher values are those with more frequent grooming and proximity associations relative to the average dyad in the community, while lower values indicated less frequent interactions. Bayesian generalised mixed models (see STAR Methods for full description of the model) were run to test the effect of social bond strength. For each model, we additionally ran pairwise comparisons between the redoings and negotiations, as well as for the respective outcomes. For the model assessing the impact of social bond strength on the use of redoings and negotiations, we additionally examined the marginal effect of social bond strength on each process. The model output showed that social bond strength had no credible effect on the probability of a redoing occurring relative to a negotiation, and their marginal effects (all 89% credible intervals spanning zero, see Table S4). In addition, no credible effect on redoing outcomes was found (all 89% credible intervals spanning zero, see Table S5). Concerning negotiations, there was, however, evidence that stronger social bonds decreased the probability of a success outcome relative to a compromise outcome (β = −0.48, 89% credible interval [CrI] [−0.87, −0.13], probability of direction [pd] = 98.54%; Figure 2; see Table S6 for full model output). There was further evidence of a community difference, with a failure outcome being higher in the Western community than in the Central community (β = 2.93, 89% CrI [0.60, 6.08], pd = 97.79%). Thus, we found partial evidence for hypothesis four that the production of redoings, negotiation, and their outcomes is influenced by the strength of the social bond between interactants, with an effect found only for negotiation outcomes.

**Figure 2:**
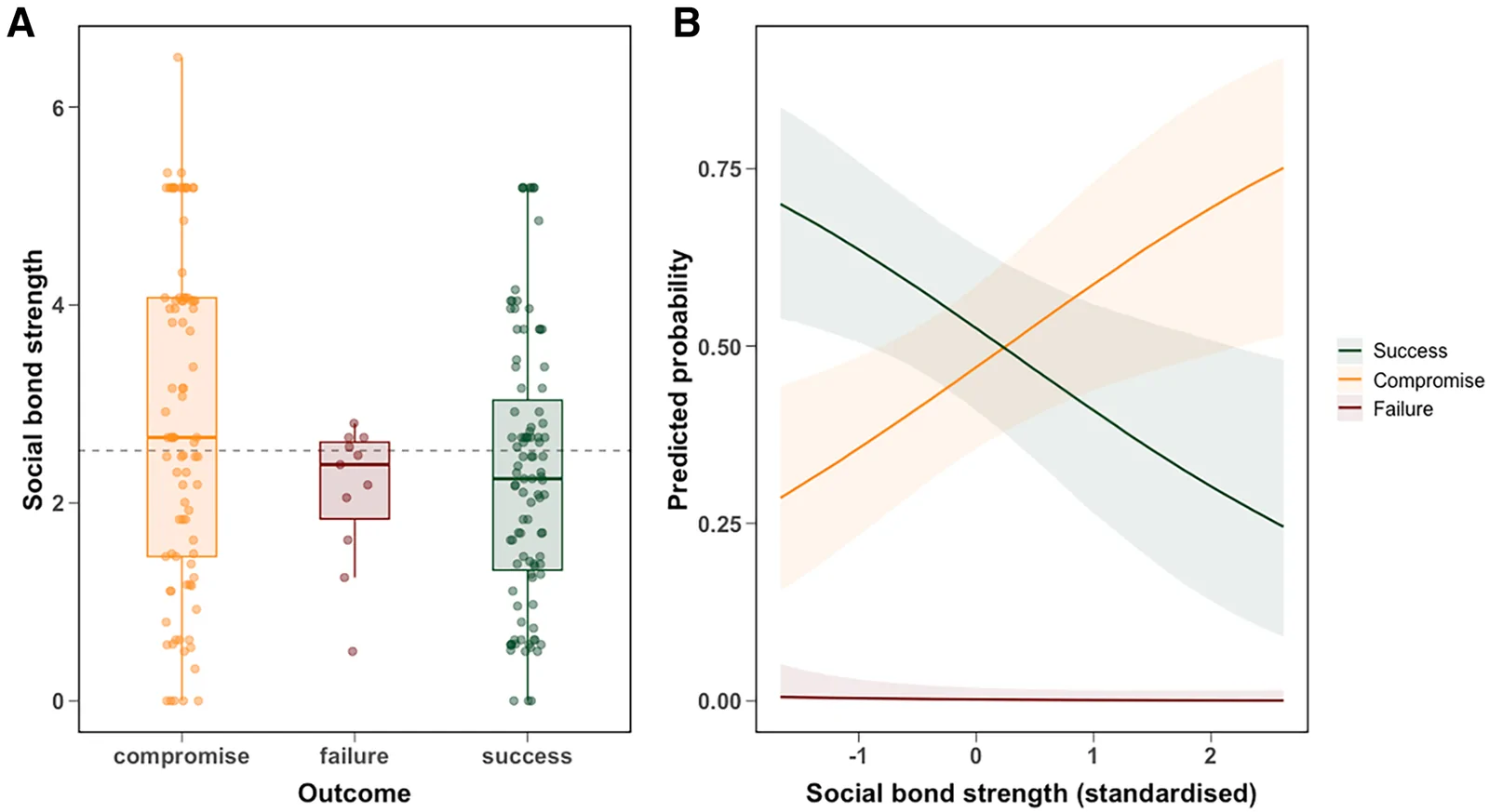
The relationship between social bond strength and negotiation outcomes excluding those with redoings (A) Distribution of observed social bond strength across each negotiation outcome (black line represents the median, box represents the 25%–75% quartile range, and whiskers represent 1.5 × the interquartile range). The points depict individual observations and the dashed line indicates the mean bond strength. (B) Predicted probability of social bond strength (standardised) on each negotiation outcome. The lines depict the posterior median predicted probability of each negotiation outcome across social bond strength, with ribbons representing 89% credible intervals derived from the model.

## Discussion

Evidence suggests that latent forms of human communicative repair may be present in other primate species (e.g.,^31^^,^^34^^,^^36^). Building on this, we examined signalling between interactants, along with how progressivity is affected during ongoing naturalistic interactions in the context of communicative breakdowns. Thus, we used male chimpanzee grooming interactions to (1) identify communicative breakdowns, (2) assess redoings and negotiations following these breakdowns and their subsequent outcomes, and (3) pinpoint similarities to human communicative repair and restoration of progressivity.

The study’s findings support the first hypothesis (H1) that redoings and negotiation represent processes comparable to human communicative repair. Structurally, chimpanzees' redoings shared certain features with human self-initiated repair, comprising repetitions, substitutions, and modifications of a previous signal following communicative failure. Communicative actions were redone or reformulated based on another participant’s behaviour, via self-monitoring, interactional contingency and flexible signalling.^12^ These findingsextend previous results on chimpanzees' communicative abilities^33^^,^^68^ by demonstrating this in a naturalistic setting and a cooperative context. Among the redoings, repetition occurred slightly more often than substitution, with modification rarely occurring. This result aligns with studies on gestural communication of zoo-housed chimpanzees, where repetition was found to be more frequent than substitution.^68^ This additionally mirrors related patterns observed in human social actions during conversations,^69^ where repetition was more common, potentially because it is cognitively economical. The low occurrence of modification in grooming interactions may reflect the focused communicative nature of this context. Grooming gestures are linked to specific interactional goals such as initiating, maintaining, or switching between grooming actions, as well as finer adjustments, including the location of grooming on the recipient’s (groomee’s) body.^53^^,^^54^^,^^56^ Breakdowns in these exchanges appear to concern the organisation of interaction rather than the structure of the gestures themselves. In such cases, repetition or substitution may offer an adequate way to re-establish coordination. In contrast, modification, involving the addition or deletion of gesture elements, may be more relevant in contexts where communicative goals or gestures used are more variable, for instance play.^70^ Hence, when gestures are strongly associated with particular goals within a certain context, altering their structure may offer little communicative advantage. This is consistent with previous work on chimpanzee signalling in captivity and the wild showing that unimodal gestures elicited a response more readily and efficiently than either other unimodal signals (facial expressions and vocalisations) or multimodal signals.^57^^,^^71^ In sum, these findings suggest that in grooming, where gesture-goal mappings are relatively stable,^53^^,^^54^ simpler gestural forms may be more effective and may require less structural adjustment through modification.

For negotiations, these may share structural features with third-turn repair. This was particularly evident in negotiations entailing two counter-signals (i.e., A gestures → B gestures → A repeats, substitutes, or modifies their original gesture). Here, gestures in the third turn were predominantly those for the same goal as their prior gestural request, suggesting that they attempted to still attain the same goal following an inadequate response from the recipient. In contrast, negotiations entailing three counter-signals (i.e., A gestures → B gestures → A gestures → B repeats, substitutes, or modifies their original gesture), while low in frequency in grooming interactions, may represent a more complex form, where both interactants engaged in flexible signalling following a response from the other. This sophisticated exchange may provide a platform from which other-prompting repair may arise. For instance, it could involve a recipient signalling communicative trouble (as seen, for example, in freeze-looks in Argentinian sign language^72^) such that the signaller can repair it,^12^ interactively distributing the repair process over participants. This is consistent with findings on conversation repair in human infants, who have been shown to produce third-turn repair from early on.^19^^,^^25^ Additionally, negotiations that included redoings, primarily repetitions and substitutions, further support the notion of an interactive platform where communicative failure can be resolved. Taken together, negotiations, ranging from one counter-signal to more extended exchanges, may provide a bilateral structural foundation from which other-initiated repair can emerge.

It is also worth noting that the higher frequency of negotiations compared to redoings may reflect the nature of breakdowns in grooming interactions of the studied male chimpanzees, which may more often involve signalling of an upcoming dispreferred response and a pursuit of a response^73^ rather than signal misrecognition. In human social actions during conversations, other-initiated repair sequences can break the continuity between a first and second pair part of an *adjacency pair* (e.g., a request and its response), halting progressivity. These can operate as pre-rejections and pre-disagreements signalling an upcoming dispreferred response.^45^ Counter-signals in chimpanzee grooming may share a similar functional role, in that they may indicate that the desired response is not forthcoming, while potentially offering both interactants the opportunity to adjust and determine the next move (e.g., who grooms whom). Although further research is needed into the context of interactional communicative exchanges, these findings offer tentative support for the proposition that other-initiated repair in humans may have roots in earlier interactional exchanges, gradually shifting toward more cooperative and linguistically mediated forms with the development of language.

In relation to temporal organisation, our results support the second hypothesis (H2) that redoings showed delayed temporal gaps that did not vary among repetitions, substitutions, and modifications. redoings occurred at longer latencies than gesture-response pairs in grooming turn-taking (median = −0.06 s^63^), suggesting contingency-driven initiation whereby a lack of timely partner response signals communicative failure and triggers a redoing. This result aligns with response waiting, a hallmark of chimpanzee intentional gestural communication in which signallers pause and maintainvisual contact with the recipient^32^^,^^74^^,^^75^ for 1 to 2 s before reinitiating.^76^^,^^77^ Although the present study did not restrict gaps to this duration, the majority of redoings fell within this time frame. This result is consistent with prior work marking this interval as the period during which the signaller monitors for a partner’s response. Longer gaps extending beyond this interval likely reflect the additional time required to produce a renewed signal or disengage from the interaction entirely. However, the temporal gaps of human communicative repair remain largely unstudied, with only one study finding delayed timing for other-initiated repair in spoken English.^22^ The question remains whether such delays are a shared feature of communicative repair across forms, languages and species.

Concerning the third hypothesis that redoings and negotiation restore progressivity (H3), we found partial support. Both repetitions and modifications often led to failure outcomes, whereas substitutions often resulted in a compromise outcome. Negotiation, by contrast, often led to compromise or success outcomes. These findings suggest that substitution and negotiation were more efficient in re-establishing progressivity. An explanation for this is that substituting one gesture for another that serves the same presumed goal may increase the likelihood of progressivity by presenting the recipient with a different signal that may be more readily responded to, without changing the underlying request. The variation in form may be sufficient to elicit a response where the original gesture did not, unlike repetitions, which reproduce the same signal and thus provide no new information to the recipient, or modifications, which alter the original signal and may therefore obscure the original request. It should be noted, however, that the low number of modifications observed limits the extent to which any conclusions can be drawn about their effectiveness. The back-and-forth nature of negotiations means both individuals remain engaged in the interaction, thereby increasing the opportunity for behavioural adjustment and ultimately progressivity. Unlike redoings, which are initiated by the signaller alone, negotiations involve the active participation of both interactants, meaning the recipient is not a passive target of the request but an active contributor to the exchange. Such exchanges may help prevent the interaction from stalling, particularly those entailing fewer counter-signals, which were the most prevalent, enabling partners to adjust their behaviour and resume grooming. This is similar to human other-initiated repair, which keeps disruption to a minimum.^13^ The sustained engagement may itself maintain the social context in which progressivity becomes more likely, and prevent further escalation toward a complete conflict of interest and interaction termination. This may be particularly important within grooming interactions given their central role in social bond maintenance in chimpanzee societies.^48^^,^^78^ In sum, beyond their structural similarities to communicative repair, redoings and negotiations of the studied male chimpanzees facilitated the re-establishment of the interaction following a communicative breakdown.

Our findings partially supported the fourth hypothesis (H4) that social bonds shape the production and outcomes of redoings and negotiation in chimpanzees. We found that the outcomes of negotiations during grooming were influenced by the social bonds of the interacting chimpanzees. In particular, a compromise outcome was more likely than a success outcome between strongly bonded pairs. This result may reflect the value of the interaction itself for strongly bonded pairs, where neither partner fully withdrew from the exchange, but instead adjusted their behaviour to reach a compromise. This flexibility allowed grooming to continue, due to its essential role in maintaining social bonds. For redoings, no effect of social bonds was found, which may reflect their signaller-driven nature. As a unilateral response to communicative failure, redoings may be less sensitive to the social relationship between chimpanzee partners than negotiation, which requires the active participation of both interactants. Social bonds in chimpanzees may therefore shape the outcomes of bidirectional processes like negotiation more than those of signaller-driven processes like redoings.

Concerning the function of grooming, we found that redoings may be more closely tied to the reciprocal and exchange-based nature of grooming.^29^^,^^51^,^52^^,^^79^ They thereby serve to maintain coordination between partners. Negotiation in male chimpanzees, in contrast, may be tied to the social bonding function of grooming,^48^^,^^65^^,^^78^ and reflect the different social pressures each dyad is facing. Weakly bonded pairs may benefit from efficient resolution that quickly restores grooming, as successful interactions may help establish or strengthen the bond. Strongly bonded pairs, in turn, may be able to afford more flexible, negotiated outcomes since the existing bond may buffer against interaction failure, allowing for the exploration of alternative responses. The way chimpanzees manage communicative breakdowns may therefore be both shaped by and in service of the broader social functions of grooming.

### Limitations of the study

While this study primarily focused on gestural communication, human communicative repair can also be multimodal in nature.^80^ Therefore, within the scope of the present study, certain processes, such as modification, may not be directly comparable to those used in human conversations. There, repair often involves the integration of speech and gestures to elaborate meaning following a breakdown in understanding by the listener.^81^ This represents a direction for future research, as incorporating a multimodal approach may increase the number of detected repair events, including modification. Individuals may substitute a gestural signal for another modality (e.g., vocalisation) or add a signal from another modality to their previous gestural signal while maintaining the same communicative intention. In addition, the recipient’s facial movements offer another domain for investigation, as these may prompt signallers to modify or repeat their signals, as shown in humans, where eyebrow furrows by a listener prompt speakers to clarify their speech.^82^

Our study additionally focused on one population and sex of East African chimpanzees, whereby the results offer insights into the communicative abilities of the studied individuals, specifically male chimpanzees. Extending this research across diverse chimpanzee populations, to female chimpanzees, sub-species and to varying social dynamics will determine whether our findings can be generalised across populations, mirroring the universality of human other-initiated repair.^6^ Similar research should be extended to other primate and non-primate species to establish phylogenetic and convergent patterns of communicative repair.

Overall, our findings showed that the chimpanzee males of the study community addressed communicative breakdowns using gestural redoings and negotiations that were primarily followed by restored progressivity. These results suggest that redoings and negotiation share structural features with human self-initiated repair and may represent early interactional building blocks,^31^ upon which more sophisticated human communicative capacities were built as language emerged,^12^ consistent with the “Interaction Engine” hypothesis.

## Resource availability

### Lead contact

Requests for further information, resources, and materials should be directed to and will be fulfilled by the lead contact, Kayla Kolff (kkolff@uni-osnabrueck.de).

### Materials availability

This study did not generate new unique reagents.

### Data and code availability

• The data are publicly available at Mendeley Data: https://data.mendeley.com/datasets/dkrwf5z9vr/1.
• The original code is publicly available at Mendeley Data: https://data.mendeley.com/datasets/dkrwf5z9vr/1.
• Any additional information required to reanalyse the data reported in this paper is available from the lead contact upon request.

## Acknowledgments

This work was funded by the German Research Foundation (DFG), project number 274877981 (DFG Research Training Group Situated Cognition, reference: GRK-2185/1), which supported K.K., and was part of an European Research Council (ERC)-Consolidator grant (772000, TurnTaking) awarded to S.P. under the European Union’s Horizon 2020 Research and Innovation Programme. We thank the Uganda Wildlife Authority, Uganda National Council of Science and Technology and the 10.13039/501100009914Makerere University for their permission to collect data for this study in the Kibale National Park. We are grateful to K. E. Langergraber, J. C. Mitani, and D. P. Watts for allowing us to study the Ngogo chimpanzee community. We express our sincere thanks to the Ngogo field team and our fellow researchers at the field site for their invaluable support both at the camp and in the field. We would like to extend a special thank you to the late S. Ramirez Amaya for his invaluable support and thought-provoking discussions. Additionally, we are especially grateful to F. Abreu, J. Vlaeyen, and R. Wilkinson for invaluable discussions or feedback on the manuscript draft. We thank the Comparative BioCognition research group for their coding assistance and administrative support. Above all, a big thank you to the Ngogo chimpanzees.

## Author contributions

Conceptualisation, K.K. and S.P.; methodology, K.K. and S.P.; data collection, K.K.; data curation, K.K.; formal analysis, K.K.; visualisation, K.K.; project administration, S.P.; supervision, S.P.; funding acquisition, S.P.; writing – original draft, K.K.; writing – review and editing, K.K. and S.P.

## Declaration of interests

The authors declare no competing interests.

## STAR★Methods

### Key resources table

**Table:** 

REAGENT or RESOURCE	SOURCE	IDENTIFIER
**Deposited data**		

Data and code for this study	Mendeley Data	https://data.mendeley.com/datasets/dkrwf5z9vr/1

**Experimental models: organisms/strains**		

*Pan troglodytes schweinfurthii* (Eastern chimpanzees)	Ngogo Chimpanzee Project, Kibale National Park, Uganda	https://ngogochimpanzeeproject.org/about-us/

**Software and algorithms**		

CyberTracker (Version 3.514)	CyberTracker Conservation 2021	https://cybertrackerwiki.org/classic/
ELAN programme (version 6.4 2022)	The Language Archive	https://archive.mpi.nl/tla/elan
EasyDIAg	ELAN programme	https://archive.mpi.nl/tla/elan
R (version 4.4.2)	R Core Team	https://www.r-project.org/

### Experimental model and study participant details

Data were collected on male chimpanzees from two communities (Central and Western) of the Ngogo population living in the Kibale National Park, Uganda. The area ranges from dry-ground and early- to mid-stage colonising forests to swamp forests and anthropogenic grasslands.^83^ The Ngogo chimpanzee population is large, including approximately 200 individuals, 116 in the Central community and 84 individuals in the Western community. We observed behavioural interactions of 42 subadult and adult male individuals (N_Central_ = 27, N_Western_ = 15) (see supplemental information, Table S1 for details). We specifically chose to collect data on males that had become independent from their mothers, which typically occurs in chimpanzees during adolescence, around the age of 13 years.^48^^,^^84^^,^^85^^,^^86^

#### Ethics statement

The research conducted on the chimpanzees was strictly non-invasive and observational, and classified as a non-animal experiment under Section V, Article 7 of the German Animal Welfare Act (25 May 1998). The study was approved by the Uganda Wildlife Authority (ref: COD/96/05), the Uganda National Council for Science and Technology (no. NS295ES), Makerere University Biological Field Station, and the Ngogo Chimpanzee Project. This research adhered to the ASAB Guidelines^87^ and the IUCN Best Practice Guidelines,^88^ which establish internationally recognised standards for ethical, non-invasive behavioural field research on animals, including nonhuman primates. These standards guided all aspects of fieldwork, including pre-fieldwork quarantine procedures, the use of surgical masks, and maintaining a distance of approximately 10 m from the chimpanzees.

### Method details

#### Data collection

Data were collected over 160 days during one consecutive field season from September 2021 to June 2022. Observations were conducted using both focal animal sampling and *ad libitum* sampling,^89^ resulting in a total of 1,064 hours of observation (Central = 698.38 hours, Western = 365.81 hours) using the CyberTracker programme on a waterproof smartphone. The mean observation time per male was 25.05 ± 10.95 hours, with focal observations ranging from ten to 41 hours. Dyadic grooming interactions during the focal follows were recorded using a digital high-definition camera (Sony FDR-AX53 4K) equipped with an external directional microphone (Sony ECM-GZ1M).

#### Study definitions

##### Gestures

This study focused on gestures that have been shown to be produced in the same^53^ and in other chimpanzee communities^54^^,^^77^ within the grooming context (see supplemental information, Table S2 for gesture details). Gestures were defined as movements of the extremities, head, or body postures that could be directed towards a social partner.^28^ They are mechanically ineffective, meaning they are produced to request or reach a goal, require active participation from the interactant, and potentially elicit a response from the recipient.^28^ The gestures showed hallmarks of intentionality^42^ (i.e. social use, response waiting, and goal-directness)^40^^,^^42^^,^^90^ as assessed through the approach of investigating communicative repair. The gestures were further categorised into a singular gesture (no overlapping gestures) or a combination of gestures (overlapping gestures), both of which were categorised as a gestural request.

##### Breakdowns, redoings, negotiation

Breakdowns were operationalised as situations within any point of a grooming interaction where a gestural request by either interacting individual did not receive an apparently satisfactory outcome^12^ within a 30s interval (time point categorising the end of a grooming interaction).^29^^,^^30^ When a gestural request was (i) not followed by an immediate response, which led to the signaller employing a redoing (*repetition*, *substitution*, or *modification*) after their initial gestural request (Figures S1 and S2), or (ii) the gestural request was counter-signalled (negotiation episode) with a gesture by the recipient instead of the signaller receiving an apparently satisfactory outcome (Figures S1 and S2). redoings were classified as *repetition*, *substitution*, and *modification*. *Repetition* involved a signaller reproducing the prior gesture or gestural sequence. *Substitution* refered to replacing a prior gesture or gestural sequence with a new one. *Modification* referred to combining another gesture with the previous gesture or reproducing the previous gestural sequence and excluding certain gestures after the failed gestural request. For assessing the REDOINGS, we only included gestures preceded by a gesture from the same signaller toward the same recipient with the same presumed goal–following established distinctions of gestural meanings in chimpanzees^53^^,^^54^–that had not received a satisfactory response.^31^ We additionally excluded cases where a response was followed immediately and refrained from applying a specific inter-event interval,^31^ as previous studies diverge regarding a temporal pause that would reflect biologically meaningful response waiting in nonhuman primates.^42^ This was further informed by the fact that responses may be anticipated before a gesture was fully completed. Communicative information may already have been conveyed at the minimum action unit (MAU, defined as the minimal movement necessary to discriminate one gesture from another),^91^ since gestures were coded from initiation to the return of the body part to a neutral state rather than from the MAU endpoint. Finally, negotiations involved the recipient producing a gesture in response to the signaller’s request, which may lead to further negotiation (signaller counter-signals, and the recipient may counter-signal again) with the possible use of redoings (Figure S1). Negotiations were further classified on the number of counter-signals (e.g., A signal - B counter-signals: 1 counter-signal; A signal - B counter-signals - A counter-signals: 2 counter-signals) and whether redoings were part of the negotiation.

##### redoing temporal gap latencies

The temporal gap latencies for the redoings were calculated from the offset of the initial or prior gestural request to the onset of the repeated, substituted, or modified gestural signal.

##### Outcomes

The outcome was examined based on whether the presumed goal (apparent satisfactory outcome) was met (see Table 3 for details) of redoings and negotiations to assess the progressivity. Responses were categorised into four outcomes based on whether the recipient’s behaviour met the presumed goal of the gestural signal: success, compromise, failure, and unclear. Success outcomes occurred when the recipient’s response met the presumed goal (for example present-groom). Compromise referred to cases where the response did not meet the presumed goal but the grooming interaction nevertheless continued (for example push-groom by signaller). In this case, the presumed goal of the push gesture is for the recipient to reposition their body, which can lead to the resumption of grooming; however, repositioning did not occur and the signaller groomed instead. Failure included three cases: no reaction, where the recipient did not react to the gesture (for example present - no response); failure, where the recipient responded but the response did not meet the presumed goal and the interaction ceased (for example present - leave); and negotiation, coded for redoings when the recipient responded with a gesture (for example present - present). Unclear was coded when the presumed goal could not be determined within the grooming context. In particular, the success and compromise outcomes represented progressivity.

#### Video coding procedure

A total of 311 dyadic grooming interactions (31.33 hours) from 157 dyads were coded using the programme ELAN (v. 6.4 2022). To establish the communicative and behavioural repertoires of grooming interactions, a template was designed based on methodological parameters^66^ and ethograms from previous studies on chimpanzees.^48^^,^^53^^,^^77^^,^^91^^,^^93^ To minimise research bias, the template was designed to gather behaviours and details without directly coding specific repair processes, repair success, and reciprocal grooming. However, these aspects could be extracted from the coded data. The template consisted of a set of tiers including “*ID*”, “*gestures*”, “*behaviours*”, and “*interaction ID*” (see Table S2, for further descriptions on gestures and behaviours). “*ID*” referred to the identity of an individual which is further separated to “*ID1*” and “*ID2”* to encompass both individuals involved in the interaction. “*Behaviours*” referred to when an individual performed social behaviours entailing approach, grooming, leaving, and repositioning. “*Gestures*” were behaviours that were classified and defined as gestures. For both “*behaviours*” and “*gestures*”, these were coded for each individual to classify which individual produced the behaviour or gesture. The *Interaction ID*” was a unique number assigned to each interaction.

Fifteen percent of all coded video clips were coded by another coder blind to the research aims, and inter-observer agreement was assessed using the EasyDIAg software package in ELAN.^94^ The system enables the testing of rater agreement in ELAN which is determined through a combined measure of the annotation value and the duration of a coded segment between raters. To reach an agreement, the duration had to overlap by at least 60% and the annotation values had to be identical.^94^ The test results indicated a “good” agreement between rater (gestures: Cohen's κ = 0.70, behaviours: Cohen's κ = 0.65) and the respective coder.

### Quantification and statistical analysis

#### Social bond strength

To determine the affiliative social relationships across dyads, a Dyadic Composite Sociality Index (DSI) was applied to determine the strength of the social relationship.^67^ The DSI score was quantified by grooming (separate from the main dataset, mean per dyad: N_Central_ = 3.03 ± 3.26, N_Western_ = 5.19 ± 5.80) and spatial proximity (mean per dyad: N_Central_ = 4.46 ± 5.01, N_Western_=7.62 ± 7.72) frequencies extracted from the scan data of the dataset. Spatial proximity patterns reflected the strength of relationships, whereas grooming patterns reflected the quality of the relationships.^65^ The DSI score was determined using the following equation:

$$\text{DSI}=\frac{\left(\frac{G_{\text{AB}}}{\bar{G}}+\frac{P_{\text{AB}}}{\bar{P}}\right)}{2}$$

G_AB_ is the rate of grooming for dyad AB and P_AB_ is the rate of proximity associations for the dyad AB. Both of each which are divided by the mean rate of the behaviour within each community. Higher values represented dyads with more frequent interactions and associations than the average dyad in their community, while lower values represented dyads with less frequent interactions and associations than the average dyad. The values ranged from 0 to 8.7484 for the Central males and 0 to 5.1822 for the Western males.

#### Statistical models

To explore whether the temporal gaps differed across redoings, we ran a Bayesian generalised mixed model. The timings were log-transformed to normalise the data and reduce heteroscedasticity, and were input as the response variable (fitting a Gaussian distribution). redoings were used as predictor variables. To test whether social bonds influenced redoings, negotiation and their outcomes, three separate Bayesian generalised mixed models were created to address this. One model included the redoings and negotiation (levels: *repetition*, *substitution*, *modification,* and negotiation – fitting a categorical distribution) as a response variable and the other two models had the outcomes (levels: success, compromise and failure – fitting a categorical distribution) as the response variables. Social bond strength was included as a predictor variable for all models. Where relevant, pairwise comparisons between levels were conducted using the “*emmeans”* package or “*hypothesis*” function, testing whether the difference between posterior distributions was credibly different from zero. Specifically for the model on social bonds and their influence on the use of redoings or negotiations, we additionally assessed the marginal effects of each type using the “avg_slopes” function.

All models were implemented in R (version 4.4.2)^95^ using the “brm” function in the *brms* package.^96^ Community identity was input as a controlled variable, and dyad and interaction identities were added as random effects. Each included four Markov chain Monte Carlo (MCMC) chains with 4000 iterations per chain and specified 1000 warm-up iterations, leading to 12000 posterior samples. For all the models, weakly informative priors were used to prevent overfitting^97^ and reduce inferential errors. For inference, we calculated 89% credible intervals (upper and lower) from the posterior distributions and checked whether zero was included in this interval.^98^^,^^99^ Herein, we considered that if posterior distributions shifted substantially away from zero in one direction, as opposed to centering on zero (i.e. which would indicate the null expectation of posterior distributions), there was evidence of an effect in a specific direction (positive or negative). The probability of direction (pd) was used to quantify the certainty of this directionality, reflecting the proportion of the posterior distribution that fell on the same side as the median estimate, with values closer to 100% indicating greater certainty that the effect was in the reported direction.

Model diagnostics were performed by examining the model summaries, which indicated that the posterior distributions accurately represented the original response values. The R-hat statistics were deemed acceptable, with values of <1.01. Furthermore, we confirmed adequate effective sample sizes (Bulk_ESS and Tail_ESS) of > 1000 and no divergent transitions in the MCMC chains. We also assessed the model convergence by visually inspecting the trace plots and density plots of the posterior distributions and checking for autocorrelation between iterations.^100^
