# Supplementary material for: When grooming stalls: Assessing communicative repair, negotiation, and progressivity in chimpanzees
Source: iScience. 2026 Sep 23;29(10):117495. doi: 10.1016/j.isci.2026.117495 (PMC13634390; doi:10.1016/j.isci.2026.117495)
Supplement: Supplementary file 1 [file mmc1.pdf]

**iScience, Volume *29***

## Supplemental information

# When grooming stalls: Assessing communicative repair, negotiation, and progressivity in chimpanzees

**Kayla Kolff and Simone Pika**

# Figures

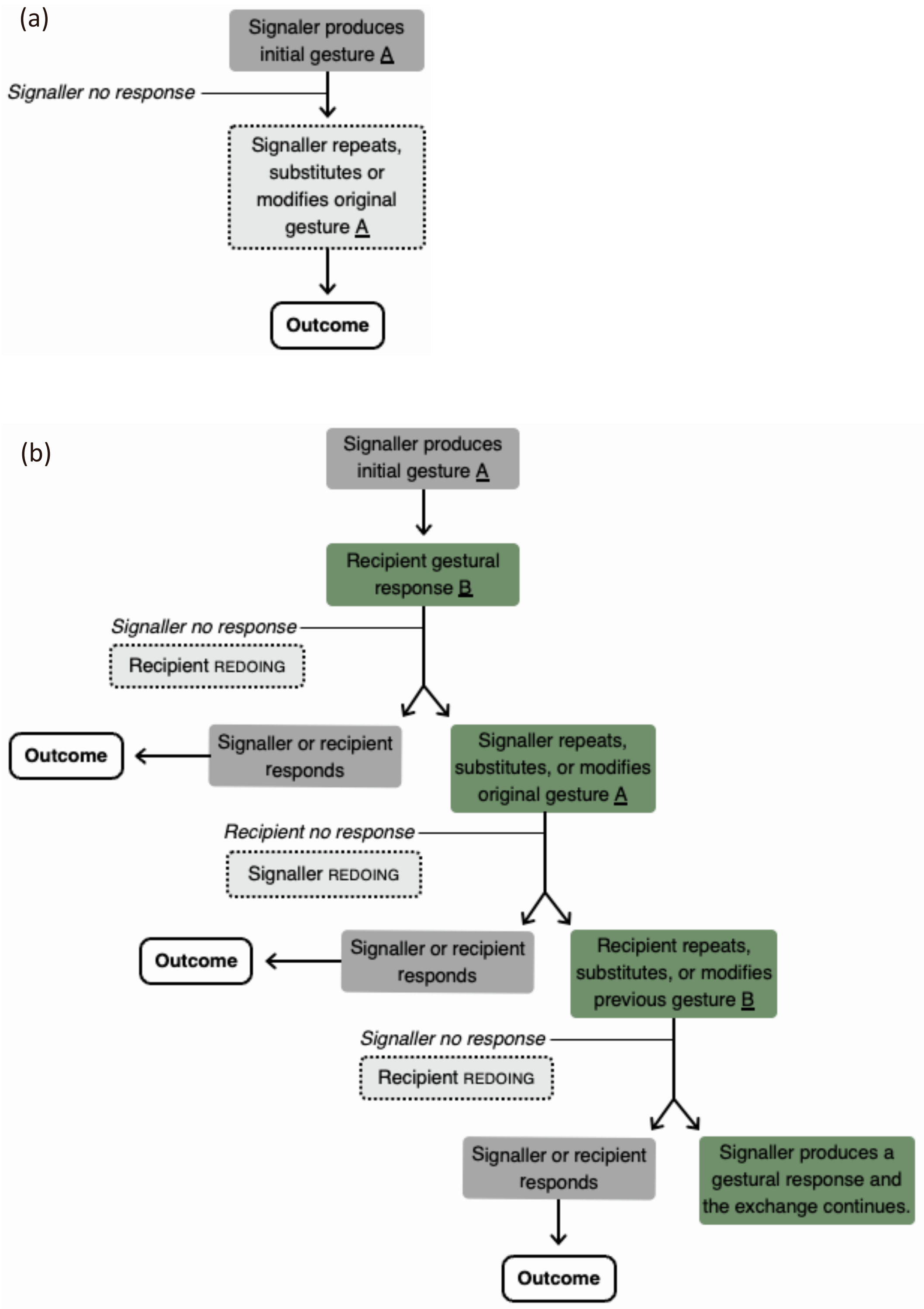


**Figure S1.** Illustration of operationalisation of (a) REDOINGS and (b) negotiation.

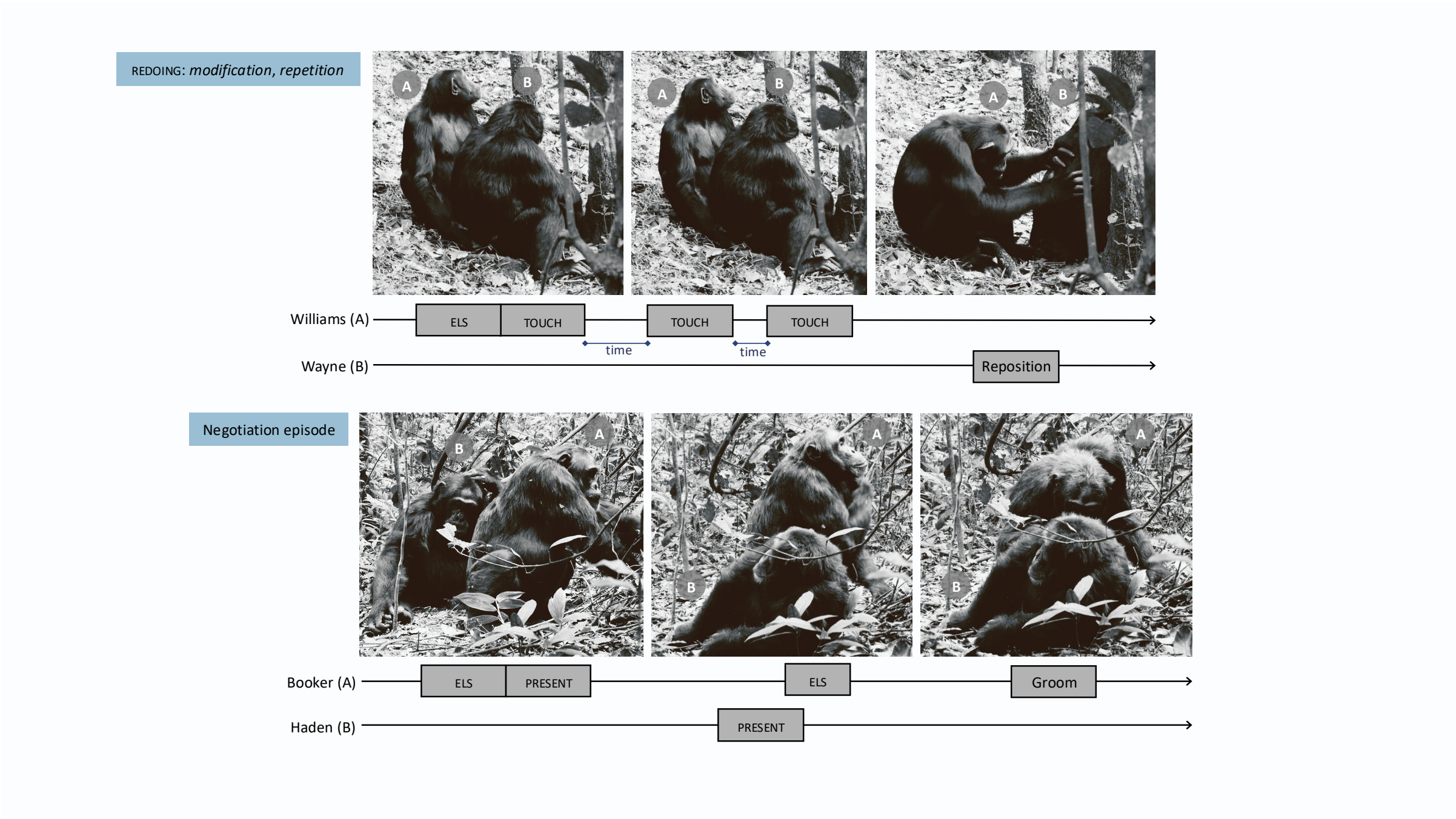


**Figure S2.** Coded example of a (a) *repetition* REDOING (See video S1) and (b) negotiation (two-counter-signals: ELS|PRESENT by A → PRESENT by B → ELS by A) without a REDOING (See video S2).

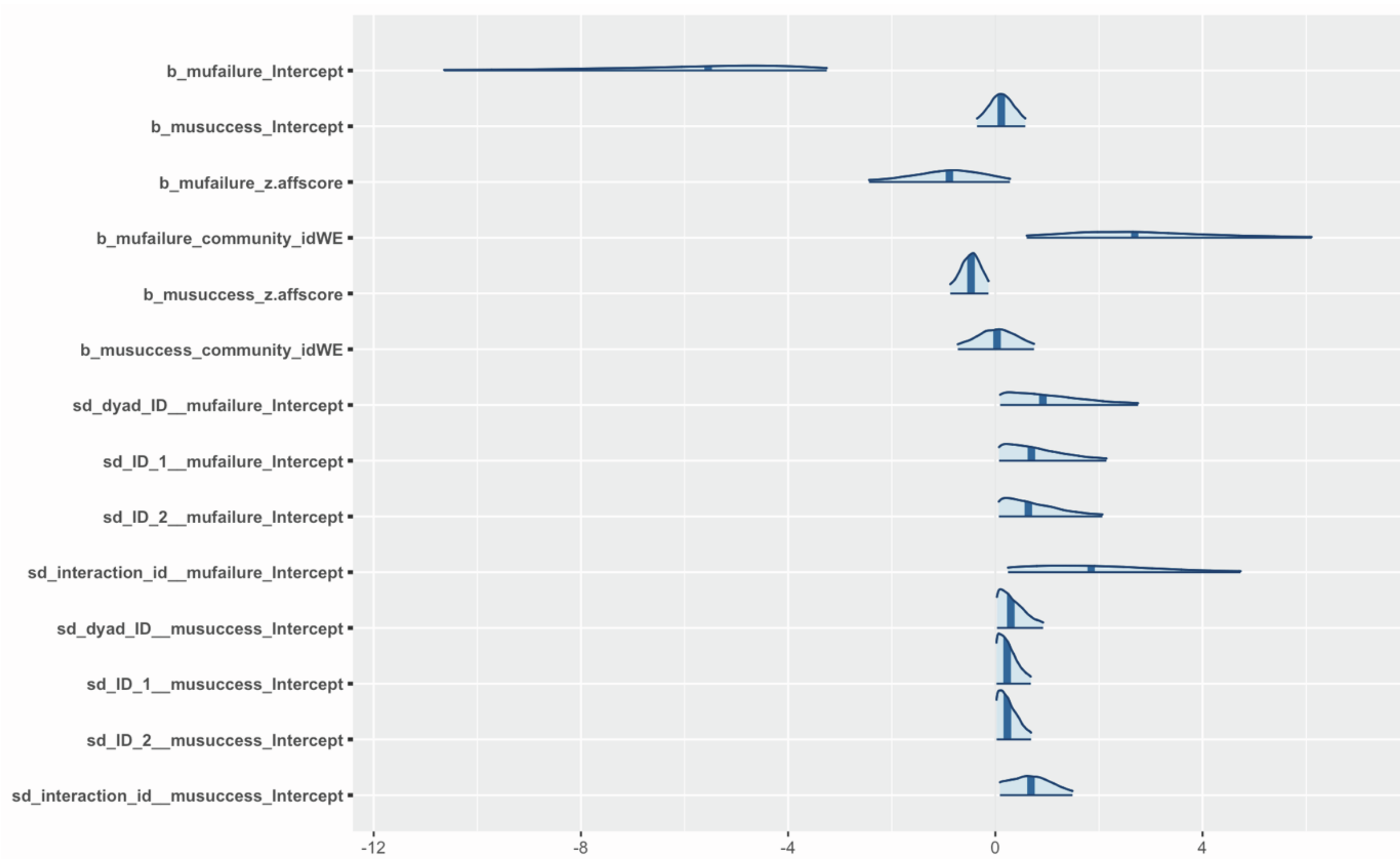


**Figure S3.** MCMC density-area plots of posterior distributions of the model 3b, testing the influence of social bond strength on the outcomes of negotiation, which had a credible effect.

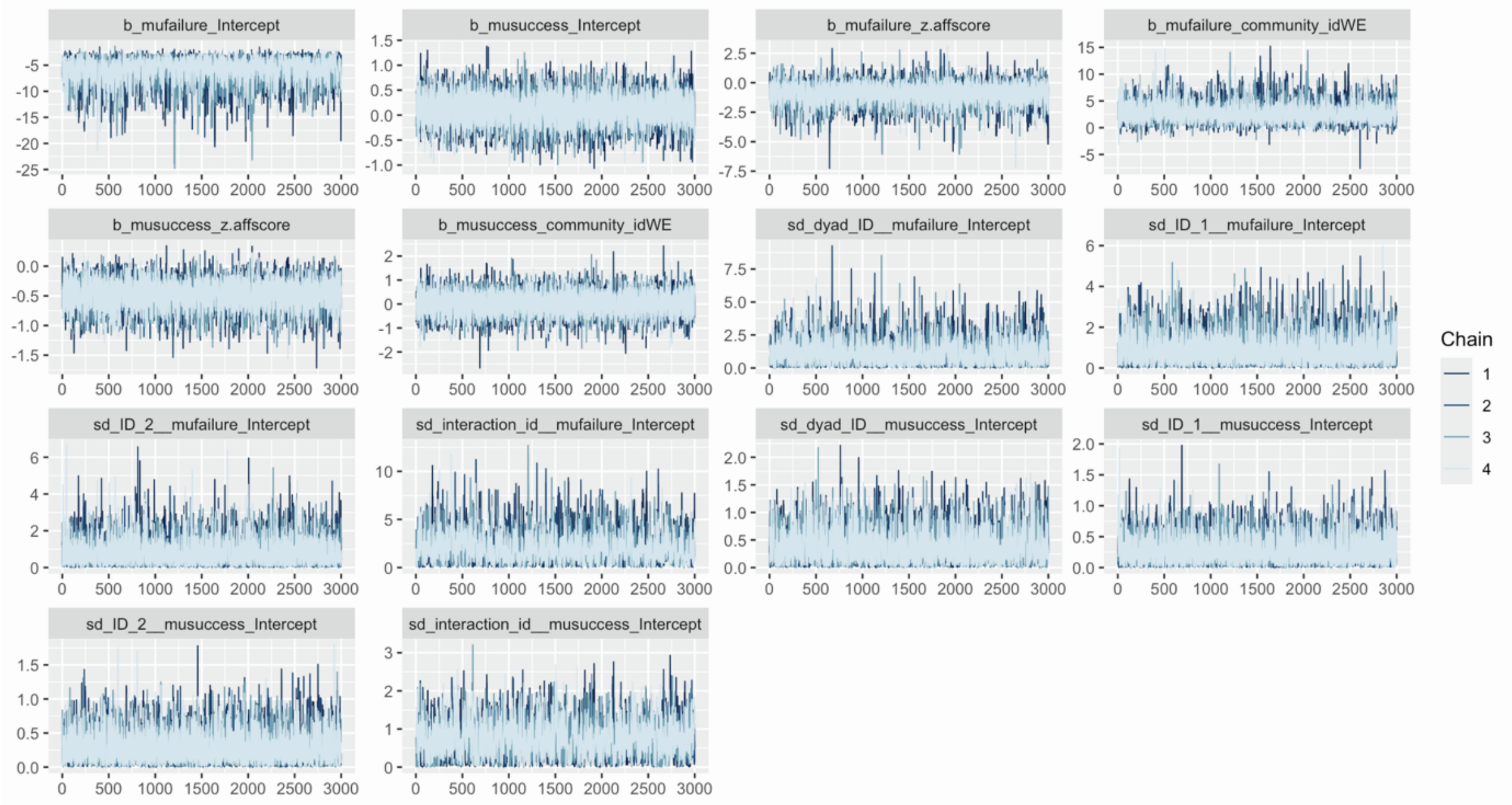


**Figure S4.** MCMC trace-plots of posterior distributions of model 3b, testing the influence of social bond strength on the outcomes of negotiation, which had a credible effect.

## Tables

**Table S1.** List of focal individuals, their age, age class, observation hours and number of REDOINGS.

| Community | Individual | Age | Age class | Observation hours | Number of REDOINGS |
|---|---|---|---|---|---|
| Central | Abrams | 22 | Adult | 30.0 | 4 |
| Central | Booker | 19 | Adult | 32.3 | 7 |
| Central | Bosko | 18 | Adult | 22.4 | 1 |
| Central | Brownface | 55 | Adult | 37.1 | 0 |
| Central | Cannonball | 14 | Subadult | 10.3 | 0 |
| Central | Carter | 30 | Adult | 35.4 | 3 |
| Central | Chopin | 21 | Adult | 31.8 | 18 |
| Central | Django | 25 | Adult | 32.5 | 0 |
| Central | Dylan | 15 | Subadult | 10.1 | 2 |
| Central | Elton | 17 | Adult | 25.1 | 5 |
| Central | Evans | 26 | Adult | 30.0 | 6 |
| Central | Fleck | 15 | Subadult | 10.5 | 4 |
| Central | Gus | 14 | Subadult | 15.7 | 1 |
| Central | Garrett | 33 | Adult | 33.1 | 8 |
| Central | Haden | 23 | Adult | 35.5 | 0 |
| Central | Jackson | 32 | Adult | 30.0 | 3 |
| Central | Miles | 41 | Adult | 36.4 | 0 |
| Central | Mitchell | 23 | Adult | 30.0 | 0 |
| Central | Morton | 38 | Adult | 32.0 | 2 |
| Central | Mulligan | 33 | Adult | 30.6 | 5 |
| Central | Nelson | 15 | Subadult | 10.5 | 0 |
| Central | Lovano | 22 | Adult | 35.6 | 1 |
| Central | PeeWee | 15 | Subadult | 14.4 | 1 |
| Central | Peterson | 26 | Adult | 35.6 | 3 |
| Central | Powel | 16 | Subadult | 10.0 | 8 |
| Central | Puccini | 13 | Subadult | 12.3 | 1 |
| Central | Wilson | 22 | Adult | 32.6 | 3 |
| Western | Bach | 13 | Subadult | 14.5 | 5 |
| Western | Buckner | 22 | Adult | 34.4 | 1 |

| | | | | | |
|---|---|---|---|---|---|
| Western | BillyBragg | 17 | Adult | 23.1 | 5 |
| Western | Damien | 15 | Subadult | 11.9 | 9 |
| Western | Freddy | 13 | Subadult | 10.2 | 0 |
| Western | Garrison | 44 | Adult | 35.8 | 5 |
| Western | Hutcherson | 28 | Adult | 33.6 | 5 |
| Western | Murray | 17 | Adult | 26.1 | 1 |
| Western | Larson | 14 | Subadult | 15.0 | 0 |
| Western | Richmond | 35 | Adult | 35.6 | 3 |
| Western | Rollins | 35 | Adult | 33.8 | 8 |
| Western | Wayne | 26 | Adult | 40.9 | 14 |
| Western | Wes | 24 | Adult | 26.9 | 2 |
| Western | Williams | 14 | Subadult | 10.0 | 9 |
| Western | Yoyo | 15 | Subadult | 14.1 | 5 |

**Table S2**. Information on coded gestures (depicted in small capitals) and behaviours identified in the study, and their definitions based on previous studies[1–9].

| Gestures | |
|---|---|
| BITE KISS | Signaller's mouth/teeth make close and gentle contact on the recipient's body which may be very brief. |
| EXAGGERATED LOUD SCRATCH (ELS) | Amplified scraping movement on signaller's own body, producing an amplified noise. If contact stops or there is a change in bodily location, it is recognised as a new ELS. |
| EXTEND LIMB | Signaller stretches their limb (usually an arm or a leg) horizontally towards or in view of the recipient. |
| GRAB-PULL | The signaller's hand (1 or 2 handed) is firmly closed over part of the recipient's body or a handful of hair and a force exerted to move the recipient’s body part towards the signaller. |
| GRAB HOLD | The signaller's hand or foot is firmly closed over a part of the recipient's body or a handful of hair, similar to GRAB-PULL but excludes the pulling and lasts for >2 seconds. |
| LEAF GROOM | A grooming indiviudal plucks a leaf and manipulates it with their fingers and/or mouth as if grooming it, that can function as an attention grabber. |

| | |
|---|---|
| PEER | The signaller maintains eye-contact with the recipient for a duration >2 seconds while being in close proximity to the recipient (within 1m). |
| PRESENT | The signaller moves their body or a body part to deliberately expose a specific area to the visual field of the recipient and the signaller maintains that position for > 2 seconds. |
| RAISE LIMB | The signaller lifts one of their body parts (typically a hand, an arm, a head, or a leg) in a generally vertical movement, often by including a brief pause of a minimum of 1 second at the peak of the movement. RAISE HAND entails only the lower part of the arm without the elbow being raised above the shoulder, while RAISE ARM involves the whole arm being raised above the shoulder. |
| PUSH | The signaller makes physical contact with the recipient's body or a body part (typically by using a hand or a foot) and applies some force to the body or the body part of the recipient. This gesture is not effective in achieving its goal (e.g., the desire that the recipient or a body part of his moves in a certain direction), but the voluntary movement is performed by the recipient to meet a specific goal. |
| TOUCH | Light and brief contact (<2 seconds) by the signaller (typically with the fingers, knuckles, hand, or foot) on the body or a body part of the recipient. |
| TOUCH HOLD | Light contact is initiated and maintained by the signaller (typically fingers, knuckles, hand, or foot) on the body or body part of the recipient with contact maintained for >2 seconds. |
| Behaviours | |
| Approach | An individual moves closer towards a recipient into a proximity within arm's length (1m). |
| Groom | Use of both hands, pushing the hair back with the thumb or index finger of one hand and holding it back while picking at the exposed skin with the nail of the corresponding thumb or index finger of the other hand. An individual can also use their mouth to hold back the hair with their lower lip while picking through the exposed skin. |
| Leave | An individual moves away from another one out of proximity. |
| Mount | An individual embraces another one with one or both arms from behind. |
| Move away | Minimal movement away from another individual and resting at a spot which is still within close proximity (arm's length, approximately 1- 2m distance) to the other individual. |
| Reposition | An individual either stays in the same spot but adjusts their bodily position, for example, by shifting from a sitting position to a laying position without exaggerating a new bodily area (otherwise classified as a PRESENT), or a slight shift (<1m) from one spot to another while maintaining close proximity (close body contact). |

**Table S3.** Model results on the influence of REDOINGS on temporal latencies. *Modification* was the reference level.

| Parameter | Estimate | Est.Error | l-89% CI | Rhat | Bulk_ESS | Tail_ESS |
|---|---|---|---|---|---|---|
| **Intercept** | **0.60** | **0.26** | **0.20, 1.01** | **1.00** | **8751** | **8677** |
| *Repetition* | 0.03 | 0.25 | -0.38, 0.43 | 1.00 | 8441 | 8188 |
| *Substitution* | -0.14 | 0.25 | -0.55, 0.27 | 1.00 | 8476 | 8280 |
| community_idWE | -0.02 | 0.14 | -0.24, 0.20 | 1.00 | 10708 | 8825 |
| *Pairwise comparison* | | | | | | |
| *Modification - Repetition* | 0.03 | - | -0.41, 0.40 | - | - | - |
| *Modification - Substitution* | 0.14 | - | -0.26, 0.55 | - | - | - |
| *Repetition - Substitution* | 0.16 | - | -0.01, 0.34 | - | - | - |

**Table S4.** Model results on the influence of social bonds on REDOINGS and negotiation. Negotiation was set as the reference level.

| Parameter | Estimate | Est.Error | 89% CI | Rhat | Bulk_ESS | Tail_ESS |
|---|---|---|---|---|---|---|
| ***Modification*: Intercept** | **-6.46** | **2.10** | **-10.11, -4.01** | **1.00** | **4939** | **3794** |
| ***Repetition*: Intercept** | **-1.69** | **0.43** | **-2.40, -1.07** | **1.00** | **7199** | **6209** |
| ***Substitution*: Intercept** | **-1.36** | **0.26** | **-1.80, -0.96** | **1.00** | **13690** | **9235** |
| *Modification*: z.affscore | -1.26 | 0.91 | -2.78, 0.00 | 1.00 | 8202 | 4969 |
| *Modification*: community_idWE | 0.72 | 1.58 | -1.64, 3.22 | 1.00 | 10026 | 6406 |
| *Repetition*: z.affscore | -0.04 | 0.26 | -0.44, 0.39 | 1.00 | 10629 | 9259 |
| *Repetition*: community_idWE | 0.04 | 0.57 | -0.86, 0.96 | 1.00 | 9966 | 8221 |
| *Substitution*: z.affscore | -0.14 | 0.18 | -0.43, 0.15 | 1.00 | 16369 | 9545 |
| *Substitution*: community_idWE | 0.36 | 0.38 | -0.24, 0.97 | 1.00 | 14187 | 8671 |
| *Average marginal effects* | | | | | | |
| *Negotiation* | 0.03 | - | -0.02, 0.08 | - | - | - |
| *Modification* | -0.02 | - | -0.05, 0.00 | - | - | - |
| *Repetition* | 0.00 | - | -0.04, 0.05 | - | - | - |
| *Substitution* | -0.01 | - | -0.06, 0.03 | - | - | - |
| *Pairwise comparison* | | | | | | |
| *Modification – Repetition* | -1.20 | 0.94 | -3.36, 0.34 | - | - | - |
| *Modification – Substitution* | -1.12 | 0.93 | -3.22, 0.42 | - | - | - |
| *Repetition – Substitution* | 0.10 | 0.30 | -0.47, 0.70 | - | - | - |

**Table S5.** Model results on the influence of social bonds on the outcomes of REDOINGS. Compromise was the reference level.

| Parameter | Estimate | Est.Error | 89% CI | Rhat | Bulk_ESS | Tail_ESS |
|---|---|---|---|---|---|---|
| Failure_Intercept | -0.68 | 0.55 | -1.62, 0.11 | 1.00 | 6948 | 6742 |
| **Success_Intercept** | **-2.72** | **1.16** | **-4.75, -1.15** | **1.00** | **4575** | **5183** |
| Failure_social bonds | -0.37 | 0.37 | -0.98, 0.21 | 1.00 | 9265 | 7479 |
| Success_social bonds | 0.24 | 0.78 | -0.95, 1.50 | 1.00 | 9380 | 7655 |
| Failure_Western community | 0.68 | 0.53 | -0.07, 1.57 | 1.00 | 6840 | 6658 |
| Success_Western community | 0.89 | 1.40 | -1.22, 3.14 | 1.00 | 5969 | 6250 |
| *Pairwise comparison* | | | | | | |
| Success – Failure | 1.06 | 0.60 | -0.02, 2.36 | - | - | - |

**Table S6.** Model results on the influence of social bonds on the outcomes of negotiation. Compromise was the reference level.

| Parameter | Estimate | Est. Error | 89% CI | Rhat | Bulk_ESS | Tail_ESS | pd |
|---|---|---|---|---|---|---|---|
| **Failure: Intercept** | **-6.05** | **2.41** | **-10.38, -3.27** | **1.00** | **2434** | **3083** | **100%** |
| Success: Intercept | 0.11 | 0.29 | -0.35, 0.58 | 1.00 | 9668 | 8237 | 64.63% |
| Failure: z.affscore | -0.97 | 0.88 | -2.49, 0.25 | 1.00 | 5650 | 4290 | 88.78% |
| **Success: z.affscore** | **-0.48** | **0.24** | **-0.87, -0.13** | **1.00** | **6906** | **6555** | **98.54%** |
| **Failure: community_idWE** | **2.93** | **1.78** | **0.60, 6.08** | **1.00** | **4048** | **4270** | **97.79%** |
| Success: community_idWE | 0.03 | 0.47 | -0.72, 0.75 | 1.00 | 9033 | 8702 | 52.50% |
| *Pairwise comparison* | | | | | | | |
| Affiliation score: Success - Failure | 0.48 | 0.89 | -1.07 2.50 | - | - | - | - |

**<u>Supplemental references to Table S2:</u>**


1. Grund, C., Badihi, G., Graham, K. E., Safryghin, A. & Hobaiter, C. GesturalOrigins: A bottom-up framework for establishing systematic gesture data across ape species. *Behav. Res. Methods* (2023) doi:10.3758/s13428-023-02082-9.
2. Goodall, J. *The Chimpanzees of Gombe: Patterns of Behaviour*. (Belknap Press of Harvard University Press, Cambridge, England, 1986).
3. Pika, S. & Mitani, J. C. Referential gestural communication in wild chimpanzees (*Pan troglodytes*). *Curr. Biol.* **16**, R191–R192 (2006).
4. Wilke, C. *et al.* Referential gestures are not ubiquitous in wild chimpanzees: alternative functions for exaggerated loud scratch gestures. *Anim. Behav.* **189**, 23–45 (2022).
5. Nishida, T., Kano, T., Goodall, J., McGrew, W. C. & Nakamura, M. Ethogram and ethnography of Mahale chimpanzees. *Anthropol. Sci.* **107**, 141–188 (1999).
6. Heesen, R., Hobaiter, C., Ferrer-I-Cancho, R. & Semple, S. Linguistic laws in chimpanzee gestural communication. *Proc. Biol. Sci.* **286**, 20182900 (2019).
7. Arcadi, A. C., Robert, D. & Boesch, C. Buttress drumming by wild chimpanzees: Temporal patterning, phrase integration into loud calls, and preliminary evidence for individual distinctiveness. *Primates* **39**, 503–516 (1998).
8. Eleuteri, V. *et al.* Chimpanzee drumming shows rhythmicity and subspecies variation. *Curr. Biol.* **35**, 2448-2456.e4 (2025).
9. van Boekholt B Bosshard AB Pika, S. Sequence organization of mother–infant interactions in chimpanzees (*Pan troglodytes schweinfurthii*) in the wild. *Proc. R. Soc. B* (2025) doi:10.1098/rspb.2025.2271.
